# The effect of breastfeeding on reducing pain induced by pentavalent vaccine in infants: a randomized clinical trial

**DOI:** 10.1590/1980-220X-REEUSP-2024-0055en

**Published:** 2024-09-09

**Authors:** Glenda Lyara Ribeiro Queiroz, Maria Augusta Rocha Bezerra, Ruth Cardoso Rocha, Mychelangela de Assis Brito, Cristianne Teixeira Carneiro, Karla Nayalle de Souza Rocha, Kaline Nayanne de Souza Oliveira

**Affiliations:** 1 Universidade Federal do Piauí, Campus Amílcar Ferreira Sobral, Departamento de Enfermagem, Floriano, PI, Brazil.; 2 Instituto Federal da Paraíba, Patos, PB, Brazil.; 3 Universidade Regional do Cariri, Crato, CE, Brazil.

**Keywords:** Breast Feeding, Vaccines, Infant, Pain, Crying, Lactancia Materna, Vacunas, Lactante, Dolor, Llanto

## Abstract

**Objective:**

To analyze the effect of breastfeeding on reducing Pentavalent vaccination pain in infants and to identify the necessary breastfeeding interval for antinociceptive action.

**Method:**

Open parallel randomized clinical trial. Ninety mother-infant dyads participated, distributed into intervention group 1 (n = 30), which breastfed five minutes before vaccination; intervention group 2 (n = 30), which breastfed five minutes before and during vaccination; and control group (n = 30), which did not breastfeed. The outcome variable was the pain level measured by the FLACC Scale. Data analysis was conducted using descriptive and inferential statistics, applying Fisher’s Exact, Kolmogorov-Smirnov, Kruskal-Wallis and Dunn’s multiple comparison tests, with 0.05 significance level.

**Results:**

Pain induced by the Pentavalent vaccine was reduced in intervention groups 1 and 2 (mean pain of 6.06 versus 3.83, respectively) compared to the control group (mean of pain of 7.43), which was significant for intervention group 2 (p < 0.001), indicating that, to achieve lower levels of pain, breastfeeding should be carried out before and during vaccination.

**Conclusion:**

Longer breastfeeding, conducted five minutes before and during vaccination, reduces the pain induced by the Pentavalent vaccine. No vaccination risks were identified to outweigh the benefits. These results endorse that health professionals should encourage breastfeeding at least five minutes before and during vaccine injection for an antinociception effect. Brazilian Clinical Trials Registry: RBR-9vh37wr.

## RESUMEN

**Objetivo:**

Evaluar el efecto de la lactancia materna en la reducción del dolor durante la vacunación pentavalente en lactantes y determinar el intervalo óptimo de lactancia para obtener un efecto antinociceptivo.

**Método:**

Ensayo clínico aleatorizado, paralelo y abierto. Participaron noventa díadas madre-lactante, divididas en grupo de intervención 1 (n = 30), que amamantó cinco minutos antes de la vacunación; grupo de intervención 2 (n = 30), que amamantó cinco minutos antes y durante la vacunación; y grupo control (n = 30), que no amamantó. La variable de resultado fue el dolor, que se evaluó utilizando la Escala FLACC. Se realizó un análisis descriptivo e inferencial de los datos, aplicando las pruebas Exacta de Fisher, Kolmogorov-Smirnov, Kruskal-Wallis y Dunn para comparaciones múltiples, con un nivel de significancia de 0,05.

**Resultados:**

El dolor inducido por la vacuna Pentavalente se redujo en los grupos de intervención 1 y 2 (dolor medio de 6,06 frente a 3,83, respectivamente) en comparación con el grupo control (dolor medio de 7,43). Esta reducción fue significativa en el grupo de intervención 2 (p < 0,001), lo que sugiere que la lactancia materna antes y durante la vacunación es más efectiva para disminuir el dolor.

**Conclusión:**

Amamantar durante cinco minutos antes y durante la vacunación pentavalente reduce el dolor inducido por la vacuna Pentavalente. No se identificaron riesgos que superen los beneficios de esta práctica. Estos hallazgos sugieren que los profesionales de la salud deben promover la lactancia materna al menos cinco minutos antes y durante la administración de vacunas inyectables para lograr un efecto antinociceptivo significativo. Registro Brasileño de Ensayos Clínicos: RBR-9vh37wr.

## INTRODUCTION

Vaccination is an asset to reduce child morbidity and mortality and has contributed to changing child health throughout history^(1)^. However, this effective public health intervention and routine pediatric practice is a common source of iatrogenic pain in childhood^(2)^.

The pain from muscle penetration by the vaccination needle is one of the first painful experiences that healthy children are faced with^(3)^, often generating concerns and fear and influencing vaccine acceptance^(4)^. Furthermore, as a private mental experience, pain is commonly latent and may go unnoticed or ignored. This undertreated, unrecognized, or poorly managed pain in childhood triggers significant and lasting negative consequences that persist in adulthood, including ongoing chronic pain, disability, and suffering^(5)^.

The prevalence of injection pain and fear of needles as barriers to vaccination varies between 5 and 13% in the general pediatric population and 8 and 28% in undervaccinated children, that is, those considered to be only partially vaccinated, with delayed vaccinations, or not vaccinated^(4)^. Consequently, needle phobia may affect 3.5% to 20% of the adult population, leading to a resistance to seeking healthcare, including vaccination^(6)^.

The lack of pain recognition and management in pediatric vaccination should not persist since evidence and methods are available for child pain management^(5)^, with an emphasis on diverse non-pharmacological measures that can provide increased analgesic options for children during vaccination^(7)^, such as distraction maneuvers, tactile stimulation, skin-to-skin contact, non-nutritive sucking, offering maternal milk, sweet solutions (25% glucose) and breastfeeding^(8)^. Out of these methods, breastfeeding stands out as an effective strategy to reduce injection pain during vaccination^(2,9)^.

Despite the benefits of the inclusion of breastfeeding as a non-pharmacological measure to reduce vaccination pain, such as decreased crying duration and heart rate, in addition to promoting the mothers’ bonds with their infants^(10)^, few professionals implement this technique in the routine of health services^(11)^. Many are still concerned that children might choke, experience bronchoaspiration, or vomit; however, there is so far no evidence in the literature identifying this phenomenon^(12)^.

Due to this context, the Brazilian Ministry of Health (MH) issued a technical note in October 2021 (Technical Note N. 39/2021)^(13)^ endorsing breastfeeding as a non-pharmacological measure to reduce pain and discomfort in children during the application of injectable vaccines. This technique is supported both by Brazilian and international studies evaluating the effectiveness of breastfeeding in reducing infant pain^(3,14)^. However, there are still gaps in the literature regarding the moment to start breastfeeding to help reduce pain when applying the vaccine, with studies evaluating breastfeeding only before^(9,15)^ and before, during, and after breastfeeding^(3,16)^, providing no comparison to identify the most suitable period for breastfeeding.

When considering that randomized and controlled studies are necessary to evaluate the efficacy and safety of breastfeeding for painful procedures^(10)^, especially the best time for intervention^(9)^ regarding how many minutes before, during and after the vaccinations^(14)^ they should be applied, the objectives of this study were to analyze the effect of breastfeeding in reducing pain induced by the Pentavalent vaccine in infants and to identify the breastfeeding time interval necessary for its antinociceptive action.

## METHOD

### Type of Study

An unblinded cluster randomized clinical trial was conducted with three parallel groups. This study’s report is based on the Consolidated Standards of Reporting Trials (CONSORT) for Randomized Trials of Nonpharmacologic Treatments and is in the Brazilian Clinical Trials Registry with primary identifier: RBR-9vh37wr.

### Local

The data were collected in vaccination rooms of the municipalities of Floriano, state of Piauí, and Barão de Grajaú, state of Maranhão, Brazil. Only Basic Health Units (BHU) in urban areas were included since vaccination rooms in the rural areas of these municipalities are not open on all working days. The 16 BHU were chosen randomly through the website www.random.org.

### Population and Selection Criteria

The population included 90 mother-infant dyads ­randomized into 3 clusters: Intervention Group 1 (IG1 - composed of dyads breastfeeding five minutes before vaccination); Intervention Group 2 (IG2 - composed of dyads breastfeeding five ­minutes before and during vaccination); and Control Group (CG - ­composed of dyads who did not breastfeed).

The inclusion criteria for mothers were being 18 years or older, currently breastfeeding (BF), with clothing suitable for breastfeeding. For infants, the following were determined: gestational age of 37–42 weeks, no congenital malformations which were visible and/or had been reported by the mother, requiring pentavalent vaccination, and aged between two months and two months and 29 days.

The exclusion criteria were infants not receiving maternal milk directly from the breast, having used painkillers in the last 48 hours before vaccination, being agitated before vaccination, having a history of hypersensitivity to any component of the immunobiological agent and/or other contraindications established by the MH^(17)^. Furthermore, for infants in the intervention groups, refusal or difficulty in breastfeeding was established as a criterion. It is emphasized that, among the groups, the infants were not required to be of the same sex, race, or weight.

### Sample Definition

The sample was calculated based on the formula for group comparison studies, considering the following parameters: significance level or type I error of α = 0.05, with 1 – α / 2 = 1.96, type II error of β = 0.1, 1 – β = 0.90, effect size or d (µ1 - µ2) = 2.3 and standard deviation (S1 = 0.4, S2 = 1.6) based on a previous study^(15)^. Based on these values, a sample size of 9 individuals was obtained for each group (control and intervention), which totaled a minimum of 27 participants as the study sample. To expand analytical capacity, data collection was continued, leading to a sample of 90 participants: 30 in IG1; 30 in IG2; and 30 in CG.

### Data Collection

Data collection was conducted by the assistant researcher from August 2022 to December 2023 in all allocated BHU simultaneously. Cluster randomization was employed; each cluster referred to one of the 16 subgroups of participants, with the inclusion of 6 in IG1, 5 in IG2, and 5 in CG. To this end, a list of the 16 subgroups was ordered in the sequence of indication provided by the Municipal Health Departments, and the numerical sequence obtained from the website was used to randomly determine which were allocated to the CG and IG. Participants were assigned to their respective groups upon their arrival for vaccination at the BHU.

During the field stage, initially conducted in a private room, guidance was provided to participating mothers about the objectives, procedures, risks, and benefits of the research. Then, an instrument was applied to characterize socioeconomic, obstetric, and aspects related to breastfeeding, used in previous studies^(18,19)^. Finally, the due specific intervention was conducted for each group in the sample. It was not possible to assign just one professional to administer the vaccines. As a result, professionals of the 16 BHU participating in the study received prior training from the team of researchers to standardize vaccination techniques and procedures.

The pentavalent vaccine was chosen for this study. This is an adsorbed vaccine for diphtheria, tetanus, pertussis, hepatitis B (recombinant), and Haemophilus influenzae type B (conjugate) presented in liquid form in multidose vials. The first of the three doses established by the Brazilian National Immunization Program was administered. This immunobiological agent is considered to be among the most painful for recipients, with the pertussis component being the main responsible for reactogenic actions, such as redness, swelling, and pain at the injection site^(20)^.

The administration technique for the pentavalent vaccine was unified for the three groups, according to the MH guidelines provided in the Manual of Vaccination Norms and Procedures (*Manual de Normas e Procedimentos para Vacinação*– MNPV)^(17)^, with an emphasis on the following aspects: the vaccine was stored between +2 ºC and +8 ºC (ideally +5 ºC), since freezing causes the formation of aggregates and increases the risk of reactions; dose volume was 0.5 ml, administered via a deep intramuscular route, into the vastus lateralis muscle of the left thigh; the needle was adapted to the administration angle according to the muscle mass of the infant to be vaccinated; a 1 ml syringe and a needle measuring 20 mm in length and 5.5 dec/mm in gauge were used.

Both IG and CG mothers held the child on their lap during the vaccination, conducted by a trained nursing professional, positioned in front of the mother. The assistant researcher was positioned laterally to the mother-infant dyad during the procedure, as per a similar previous study^(3)^.

During pentavalent vaccination the validated FLACC behavioral scale was applied. This scale was developed to assess pain in children between two months and seven years old^(21)^. The scale presents five assessment categories according to the meaning of the initials of the scale: Face, Legs, Activity, Cry, and Consolability. Each category can be scored on a scale of zero to two and has a result ranging from 0–10, in which zero is considered relaxed or comfortable, 1–3 means minor discomfort, 4–6 moderate pain and 7–10 severe discomfort. In 2008, Silva and Thuler^(22)^ translated and culturally adapted the scale into Brazilian Portuguese, obtaining satisfactory results.

### Intervention

The main outcome variable was pain reduction in ­vaccinated infants. As a secondary outcome variable, the best necessary time interval for breastfeeding (only before or before and during vaccination) for antinociception action was determined. The intervention was conducted in both IG1 and IG2. For the ­mother-infant dyads allocated to IG1, mothers were asked, still in the private room and in a comfortable chair, to breastfeed the infant for 5 minutes before vaccination, which was monitored using a stopwatch. It was emphasized that monitoring would begin as soon as there was proof that the infant was sucking effectively based on the key points established by the MH for determining adequate latch: 1. More areola visible above the baby’s mouth; 2. Mouth wide open; 3. Lower lip turned outward; 4. Chin touching the breast. Visible and/or audible swallowing^(23)^ was also identified, which indicates nutritive sucking, i.e., that the infant was swallowing breast milk. Then, mothers were advised to suspend breastfeeding during vaccination.

For the mother-infant dyads allocated to IG2, mothers were asked, still in the private room and in a comfortable chair, to breastfeed their infant for 5 minutes before vaccination, according to the criteria established for IG1. Subsequently, mothers were advised to suspend breastfeeding only while the Human Rotavirus Vaccine was administered, following the guidelines of the Brazilian MH, which determines that oral vaccines should be administered before injectable vaccines^(13)^. They were immediately instructed to restart breastfeeding and maintain it throughout pentavalent vaccination, which was ended with gentle compression at the vaccine site with dry cotton. The data for the intervention groups (IG1 and IG2) was collected in approximately 30 minutes.

The participants allocated to the CG were mother-infant dyads receiving the usual care from the health service and, therefore, without conducting breastfeeding before or before and during vaccination. The duration of the CG data collection was approximately 15 minutes. All participants then received a printout with Technical Note N°39/2021 from the MH.

### Data Analysis and Treatment

The data were double-entered and stored in Microsoft Excel® version 2011 spreadsheets. They were then processed and analyzed in the statistical program Package for Social Sciences for Windows (SPSS) (2009) version 20.0. A descriptive analysis of the data was conducted using absolute and relative frequencies, as well as the measure of central tendency, mean, median, and standard deviation. To verify the homogeneity of the data in the IG and the CG, the Fisher’s Exact test was employed for qualitative variables. The distribution of outcome variables was then assessed using the Kolmogorov-Smirnov test. Since its distribution was not normal, the results of non-parametric tests, such as the Kruskal-Wallis test and Dunn’s multiple comparisons test, were reported for group and pairwise comparisons, respectively. A significance level of 5% and a 95% confidence interval were adopted for all tests.

### Ethical Aspects

The study complied with Resolutions No. 466/12 and No. 580/2018 of the National Health Council. It was approved by the Research Ethics Committee of the Amílcar Ferreira Sobral Campus of Universidade Federal do Piauí in 2023 under Opinion no. 6.083.435. Participation depended on the participants’ signing of the Informed Consent Form.

## RESULTS

**Figure 1:**
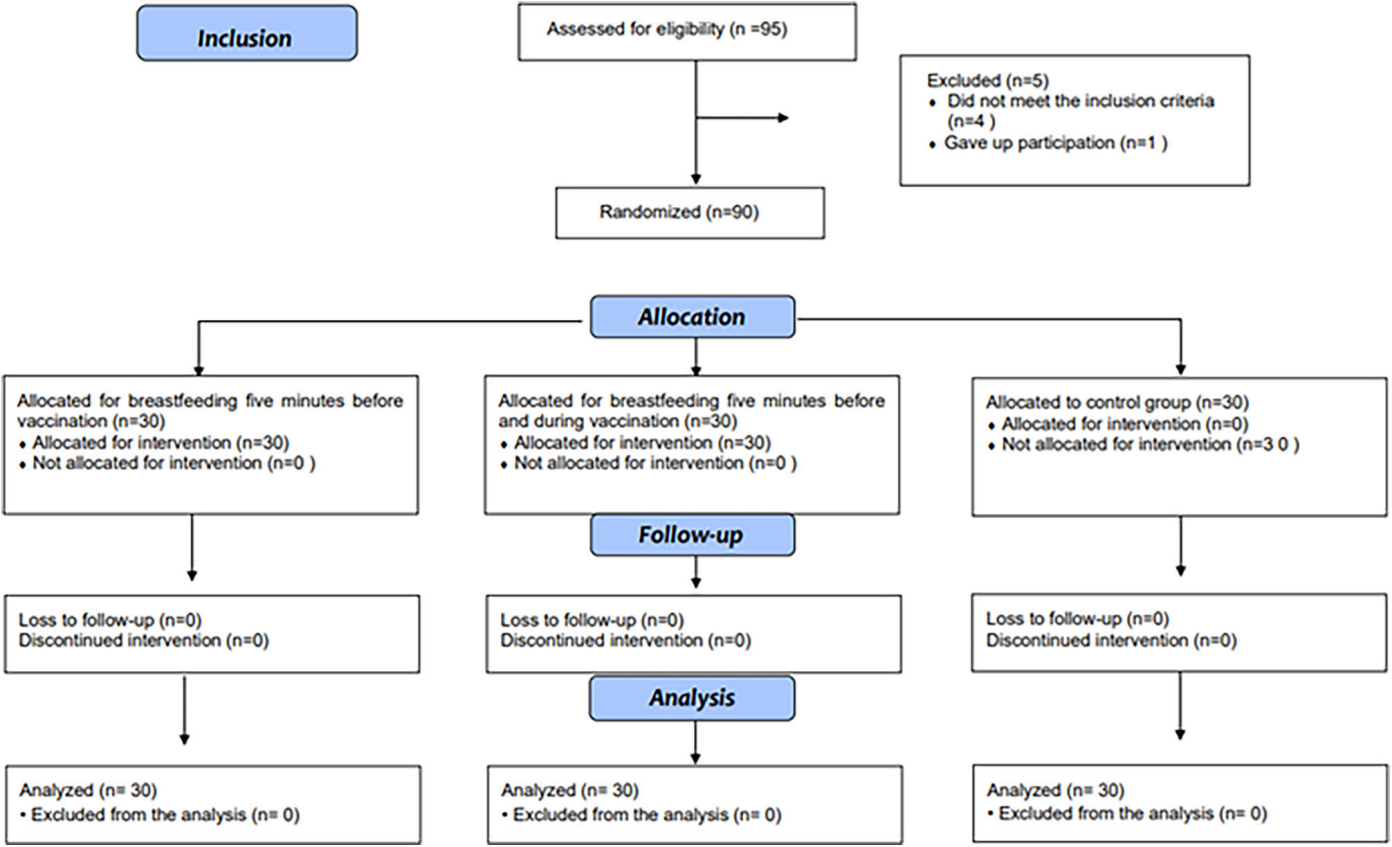
Diagram representing the flow of participants in each phase of the study, adapted from the Consolidated Standards of Reporting Trials (CONSORT). Floriano, state of Piauí/Barão de Grajaú, state of Maranhão, Brazil, 2022-2023 (n = 90).

Ninety-five mother-infant dyads were eligible for evaluation. Five of them did not meet the inclusion criteria and thus ninety were randomized into three groups: breastfeeding before vaccination (30 participants), breastfeeding before and during vaccination (30 participants) and control (30 participants). The flowchart for tracking participants included in the study is shown in Figure 1.

There was no statistically significant difference between the intervention and control groups in terms of socioeconomic characteristics age group, ethnicity, education, marital status, family income, and performing paid work (p > 0.05); similarly, the obstetric profile did not differ significantly between the groups regarding gestational age, prenatal consultation, breastfeeding guidance, pregnancy complications, and type of delivery (p > 0.05). The groups were also homogeneous regarding aspects related to the breastfeeding process: whether the infant was breastfed immediately after birth and whether there was ­skin-to-skin contact at birth (p > 0.05) (Table 1).

**Table 1:** Characterization of mothers regarding socioeconomic, obstetric, and breastfeeding data in the control and intervention groups. Floriano, PI/Barão de Grajaú, MA, Brazil 2022/2023 (n = 90).

Variables	Intervention Group 1 n* (%)	Intervention Group 2 n* (%)	Control Group n* (%)	Mean ± SD^†^	p-Value^‡^
	Socioeconomic characterization				
**Age Range**				28.48 ± 6.63	0.273^§^
18 to 28 years old	18 (60.0%)	17 (56.7%)	12 (40.0%)		
29 to 43 years old	12 (40.0%)	13 (43.3%)	18 (60.0%)		
**Ethnicity**					0.206^§^
White	5 (16.7%)	1 (3.3%)	1 (3.3%)		
Brown/Black	25 (83.3%)	29 (96.7%)	29 (96.7%)		
**Education**					0.954^§^
Incomplete Elementary Education to Incomplete Secondary Education	7 (23.3%)	8 (26.7%)	9 (30.0%)		
Complete Secondary Education to Complete Higher Education	23 (76.7%)	22 (73.3%)	21 (70.0%)		
**Marital status**					0.070^§^
Single	8 (26.7%)	14 (46.7%)	6 (20.0%)		
Married	22 (73.3%)	16 (53.3%)	24 (80.0%)		
**Family income** ^¶^					0.285^§^
Up to 2 minimum wages	27 (90.0%)	22 (73.3%)	26 (86.7%)		
>2 minimum wages	3 (10.0%)	8 (26.7%)	4 (13.3%)		
**Performing paid work**					0.421^§^
No	20 (66.7%)	18 (60.0%)	23 (76.7%)		
Yes	10 (33.3%)	12 (40.0%)	7 (23.3%)		
**Obstetric characterization**					
**Gestational age**				39.03 ± 1.35	0.184^§^
37**–**39	20 (66.7%)	16 (53.3%)	23 (76.7%)		
40**–**42	10 (33.3%)	14 (46.7%)	7 (23.3%)		
**Prenatal consultation**	**–**
No	**–**	**–**	**–**		
Yes	30 (100%)	30 (100%)	30 (100%)		
**Received guidance on breastfeeding**					0.554^§^
No	8 (26.7%)	6 (20.0%)	10 (33.3%)		
Yes	22 (73.3%)	24 (80.0%)	20 (66.7%)		
**Had complications during pregnancy**					0.114^§^
No	28 (93.3%)	22 (73.3%)	22 (76.7%)		
Yes	2 (6.7%)	8 (26.7%)	7 (23.3%)		
**Type of birth**					0.876^§^
Cesarean	22 (73.3%)	22 (73.3%)	20 (66.7%)		
Normal	8 (26.7%)	8 (26.7%)	10 (33.3%)		
**Infant was breastfed immediately after birth**					0.451^§^
No	14 (46.7%)	12 (40.0%)	9 (30.0%)		
Yes	16 (53.3%)	18 (60.0%)	21 (70.0%)		
**There was skin-to-skin contact at birth**					0.774^§^
No	16 (53.3%)	13 (43.3%)	13 (43.3%)		
Yes	14 (46.7%)	17 (56.7%)	17 (56.7%)		

*n = sample;

^†^SD = Standard deviation;

^‡^ *p-*Value = significance level;

^§^Fisher’s exact test;

^¶^Current minimum wage = R$1,212, Brazil, 2022.

The average pain for the infants in IG1 (breastfed only before vaccination) was 6.06 ± 1.25 (Median = 6); in IG2 (breastfed before and during vaccination), the average pain was 3.83 ± 1.23 (Median = 4); and in the control group, the average pain level of the infants was 7.43 ± 1.30 (Median = 7.5). Paired comparisons showed that there is an effect of breastfeeding on pain reduction [X^2^(2) = 52.238; p < 0.05) among groups (Table 2).

**Table 2:** Comparative analysis between groups using the FLACC scale. Floriano, PI/Barão de Grajaú, MA, Brazil, 2022/2023 (n = 90).

Pain score – FLACC scale									p-Value^1^
Intervention group I (n=30)			Intervention group II (n = 30)			Control group (n = 30)			0.000
Min	Max	Median (IQR) / Mean ± SD	Min	Max	Median (IQR) / Mean ± SD	Min	Max	Median (IQR) / Mean ± SD	
4	9	6 (2) / 6.06 ± 1.25	2	7	4 (1) / 3.83 ± 1.23	5	10	7.5 (2.25) / 7.43 ± 1.30	

^1^Kruskal-Wallis.

As previously mentioned in the data analysis subsection, we performed a post hoc analysis using Dunn’s multiple comparisons test considering the Bonferroni Correction equal to 0.0167. First, in both tests, IG1 and IG2 were compared with the control group in behavioral pain responses during pentavalent vaccination. The results showed that only IG2 presented a significantly (p < 0.05) lower score in behavioral pain responses compared to the control group. In other words, breastfeeding 5 minutes before and during vaccination was more effective than breastfeeding only before in decreasing infants’ behavioral pain responses during pentavalent vaccination (Table 3).

**Table 3:** Comparative analysis of pairs of groups using the FLACC scale. Floriano, PI/Barão de Grajaú, MA, Brazil, 2022/2023 (n = 90).

Sample 1 - Sample 2	Statistical test	Standard deviation	p-Value^1^	Intergroup p-Value^1^
IG2 - IG1	29.567	6.67	0.000	0.000
IG2 - CG	47.783	6.67	0.000	0.000
IG1- CG	18.217	6.67	0.006	0.019

^1^Dunn’s Multiple Comparisons Test.

Pain classification was also evaluated considering the investigated groups, as described in Table 4. No infant in IG1 had mild pain; 20 had moderate pain and 10 had strong pain. In IG2, 14 infants had mild pain, 14 had moderate pain, and only 2 had strong pain. Finally, in the control group, none of the infants had mild pain, while 8 had moderate pain and 22 had strong pain (p = 0.000).

**Table 4:** Comparative analysis of pain classification. Floriano, PI, Brazil, 2023.

Intervention group I			Intervention group II			Control group			p-Value^1^		
Mild	Moderate	Strong	Mild	Moderate	Strong	Mild	Moderate	Strong	**0.000**
**–**	20	10	14	14	2	**–**	8	22	

^1^Fisher’s exact test.

## DISCUSSION

The results demonstrated that infants in IG2 (breastfed with nutritive sucking, that is, swallowing breast milk, five minutes before and during the administration of the pentavalent vaccine) obtained a better behavioral response to reduce pain, observed using the FLACC scale, when compared to the other groups. Although pain among IG1 infants (breastfed only before vaccination) was reduced, discomfort levels were still high.

The average pain score for IG2 was 3.83 (±1.23), while for IG1 and CG it was 6.06 (±1.25) and 7.43 (±1.30), respectively. The difference was statistically significant (p = 0.001) only in IG2, which indicates that, to significantly reduce pain in infants during vaccination, breastfeeding needs to be conducted at an opportune time.

The indication of the antinociceptive action of breastfeeding in this study adds to the body of literature that supports this practice during routine procedures, such as vaccination, to reduce pain among infants. A scoping review aimed at examining how research on non-pharmacological management of children with vaccination pain in the healthcare setting was conducted recommended, as a first alternative, breastfeeding, then sweetened solutions and, finally, non-nutritive sucking to reduce vaccination pain in newborns and infants^(7)^.

Based on the evidence presented in Technical Note n. 39/2021^(13)^ about non-pharmacological interventions to reduce vaccination pain in breastfed infants, health services are recommended to encourage and support the presence parents or guardians during and after vaccination and encourage the nursing mother to breastfeed the child immediately before and during the administration of injectable vaccines.

Regarding the appropriate time for breastfeeding initiation and duration, the results are similar to those of a study conducted with the objective of determining breastfeeding effectiveness for pain relief during the vaccination of babies breastfed two minutes before and during the procedure; such study demonstrated that breastfeeding significantly reduced pain levels^(24)^. A study aimed at identifying the effect of breastfeeding on the intensity of immunization pain in infants breastfed before, during and after vaccination also concluded that breastfeeding has a highly expressive, statistically significant positive effect as a non-pharmacological method in reducing pain intensity among infants^(16)^.

On the other hand, in a study aimed at investigating the effectiveness of breastfeeding in reducing pain in newborns undergoing the heel prick test, the researchers argued that there was no significant difference in the mean pain scores during heel blood collection after breastfeeding in the study and control groups; they acknowledged the possibility that the time interval (two minutes before, with the interruption of breastfeeding prior to the painful procedure) was not long enough to obtain the antinociceptive effect of the breast milk^(25)^.

Breastfeeding infants five minutes before and during vaccination is thus sufficient to reduce pain. This time interval that was also established in previous studies involving the administration of the hepatitis B^(12)^ and conjugated pneumococcal^(14)^ vaccines, in which there was a reduction in pain, converging with the results of this research.

Breastfeeding provides better behavioral responses to pain, reducing crying time and pain scores, during vaccination compared to no intervention, drinking water, and other interventions, such as cuddling, oral glucose intake, topical anesthetic agents, massage, and cooling sprays^(10)^. In breastfeeding a complex network of multifactorial components is integrated, allowing the maximum reach of the analgesic capacity of this practice. It is inferred that, from the moment the mother is prepared, when she places the infant on her lap to allow the beginning of non-nutritive sucking, which is responsible for triggering the milk ejection reflex, until nutritious sucking is achieved, chemical and behavioral phenomena converge to generate relaxation and pain relief for the infant.

The mechanisms underlying the beneficial effect of breastfeeding against vaccination pain are still undefined^(3)^. However, a previous study found that due to the sweetness of sucrose present in human milk and the oral and tactile stimulation of non-nutritive sucking, serotonin and endorphin are released, producing an analgesic effect that lasts from five to ten minutes^(26)^. This fact is associated with the stimulation of the infants’ senses through maternal scent^(27)^, heartbeat listening^(28)^, and the tactile sensation of containment and protection promoted by the mother’s lap^(29)^.

A review in the Cochrane database aimed at evaluating the effectiveness of breastfeeding or breast milk supplementation in pain reduction among neonates indicated that possibly analgesic components of breastfeeding include the presence of a comforting person (mother), physical sensation (skin-to-skin contact with the comforting person), diversion of attention/distraction and sweetness of human milk (presence of lactose or other components)^(10)^.

This combination of mechanisms, which suggest breastfeeding’s potential for reducing vaccination pain, explains the results of this study, which indicate breastfeeding only before vaccination as an insufficient antinociceptive agent. Therefore, different non-pharmacological interventions might have coordinated analgesic effects and the combination provided by breastfeeding is recommended to maximize analgesia during vaccination^(7)^.

Other non-pharmacological methods can be used for pain management in non-breastfed infants undergoing painful procedures, which can be applied alone or in combination, namely: oral administration of sweet solutions, such as sucrose, glucose and dextrose, in different concentrations; non-nutritive sucking; Kangaroo Mother Care and skin-to-skin contact; swaddling; application of mechanical vibration; massage; containment; cuddling position; among others.

Among the infants breastfed during vaccination, none presented complications, such as choking, coughing, aspiration, or cyanosis. Only one child in IG1 regurgitated after administration of the human rotavirus vaccine, a condition that may be associated with the vaccine itself^(17)^. The belief in the possibility of these complications, often verbalized by nursing professionals, is limiting and can have a negative impact by discouraging breastfeeding during vaccination to relieve pain in newborns and infants^(30)^. In a systematic review of the Cochrane database, none of the included studies reported complications related to breastfeeding during invasive procedures, thus suggesting that there is no risk of adverse effects such as those mentioned^(31)^.

This study has relevant results for health professionals when considering that breastfeeding constitutes a natural intervention and does not require special facilities or financial investments. Therefore, this non-pharmacological method should be implemented in vaccination rooms for pain management and control in infants. It is up to health professionals, with an emphasis on nursing teams working in primary health care services, especially vaccination rooms, to encourage the practice of breastfeeding during painful procedures, such as the administration of injectable vaccines. Finally, pain assessment scales are recommended to be used by the nursing team in the routine of vaccination rooms as instruments to evaluate the quality of experiences and the efficiency of their approaches.

This is a single-center study, whose external validity is restricted to one region of Brazil. A subsequent multicenter study with a larger sample is needed to confirm this study’s results. Another limitation is the fact that, due to the nature of the interventions, blinding the team members who conducted them and the participants was not possible. Furthermore, the application of the FLACC scale, which assesses behavioral responses, constitutes another limitation, although it is validated. Thus, an evaluation of objective parameters, such as physiological measurements, is suggested for future studies. Despite these limitations, the analysis proved the intervention to be effective.

## CONCLUSION

Breastfeeding five minutes before and during the administration of the pentavalent vaccine significantly reduced the pain score when compared to breastfeeding just before the procedure. No risks were identified that could outweigh the benefits of breastfeeding during vaccination; therefore, this practice must be implemented in the routine of vaccination rooms, as it is a natural, accessible, and feasible method. Therefore, Technical Note n. 39/2021 from the Brazilian Ministry of Health is endorsed, while it is suggested that the term “immediately”, present in this document, should correspond to five minutes before vaccination.
