# Supplementary material for: The effect of breastfeeding on reducing pain induced by pentavalent vaccine in infants: a randomized clinical trial
Source: Rev Esc Enferm USP. 2024 Sep 9;58:e20240055. doi: 10.1590/1980-220X-REEUSP-2024-0055en (PMC11391782; doi:10.1590/1980-220X-REEUSP-2024-0055en)
Supplement: Translation

# Translation: Efeito da amamentação na redução da dor induzida pela vacina Pentavalente em lactentes: ensaio clínico randomizado

**DOI:** 10.1590/1980-220X-REEUSP-2024-0055pt

**Authors:** Glenda Lyara Ribeiro Queiroz, Maria Augusta Rocha Bezerra, Ruth Cardoso Rocha, Mychelangela de Assis Brito, Cristianne Teixeira Carneiro, Karla Nayalle de Souza Rocha, Kaline Nayanne de Souza Oliveira

**Affiliations:** 1 Universidade Federal do Piauí, Campus Amílcar Ferreira Sobral, Departamento de Enfermagem, Floriano, PI, Brasil.; 2 Instituto Federal da Paraíba, Patos, PB, Brasil.; 3 Universidade Regional do Cariri, Crato, CE, Brasil.

**Keywords:** Aleitamento Materno, Vacinas, Lactente, Dor, Choro

## Abstract

**Objetivo:**

Analisar o efeito da amamentação na redução da dor induzida pela vacina Pentavalente em lactentes e identificar o intervalo de tempo da amamentação necessário para sua ação antinocicepção.

**Método:**

Ensaio clínico randomizado paralelo aberto. Participaram 90 binômios mãe-lactente, distribuídos em grupo intervenção 1 (n = 30), que realizou a amamentação cinco minutos antes da vacinação; grupo intervenção 2 (n = 30), realizou a amamentação cinco minutos antes e durante a vacinação; e grupo controle (n = 30), que não realizou a amamentação. A variável desfecho foi o nível de dor mensurado pela Escala FLACC. A análise dos dados foi realizada por meio de estatística descritiva e inferencial, com aplicação dos testes Exato de Fisher, Kolmogorov-Smirnov, Kruskal-Wallis e de comparações múltiplas de Dunn, adotando nível de significância de 0,05.

**Resultados:**

A dor induzida pela vacina Pentavalente se reduziu nos grupos intervenção 1 e 2 (média de dor de 6,06 versus 3,83, respectivamente) em comparação ao grupo controle (média de dor de 7,43), o que foi significativo para o grupo intervenção 2 (p < 0,001), indicando que, para alcançar menores níveis de dor, a amamentação deve ocorrer antes e durante a vacinação.

**Conclusão:**

A amamentação mais prolongada, realizada cinco minutos antes e durante todo o processo de vacinação, reduz a dor induzida pela vacina Pentavalente. Em sua aplicação não foram identificados riscos capazes de superar os benefícios de tal prática. Esses resultados endossam a importância de os profissionais de saúde incentivarem essa prática no tempo mínimo de cinco minutos antes e durante a aplicação de vacinas injetáveis para obtenção do efeito antinocicepção. Registro Brasileiro de Ensaios Clínicos: RBR-9vh37wr.

## INTRODUÇÃO

A vacina é um dos mecanismos adotados para a redução da morbimortalidade infantil e tem contribuído para modificar a situação de saúde das crianças ao longo da história^(1)^. No entanto, embora seja uma intervenção eficaz de saúde pública e uma prática pediátrica de rotina, é uma fonte comum de dor iatrogênica na infância^(2)^.

A dor gerada durante a perfuração do músculo pela agulha na vacinação é uma das primeiras experiências dolorosas que as crianças saudáveis precisam enfrentar^(3)^, com possibilidade de gerar preocupações e medo e influenciar a aceitação da vacina^(4)^. Ademais, como uma experiência mental privada, a dor muitas vezes fica oculta e pode passar despercebida ou ser ignorada. Essa dor subtratada, não reconhecida ou mal gerida na infância desencadeia consequências negativas importantes e duradouras que continuam na idade adulta, incluindo a dor crônica contínua, incapacidade e sofrimento^(5)^.

A prevalência da dor causada pela injeção e do medo de agulhas como barreiras à vacinação varia entre 5 e 13% na população pediátrica geral e 8 e 28% na subvacinada, ou seja, crianças consideradas parcialmente vacinadas, com vacinas atrasadas ou não vacinadas^(4)^. Como consequência, a fobia de agulhas pode chegar a afetar de 3,5% a 20% da população adulta, implicando a resistência pela busca por cuidados de saúde nessa fase, incluindo vacinas^(6)^.

A falta de reconhecimento e gestão da dor na vacinação pediátrica não deve continuar, especialmente porque existem evidências e métodos para o manejo da dor infantil^(5)^, com destaque para medidas diversificadas não farmacológicas que podem fornecer mais opções analgésicas para as crianças durante a vacinação^(7)^, tais como manobras de distração, estimulação tátil, contato pele a pele, sucção não nutritiva, oferta de leite materno, soluções adocicadas (glicose 25%) e amamentação^(8)^. Dentre esses métodos, a amamentação destaca-se como uma estratégia eficaz para reduzir a dor da injeção durante a vacinação^(2,9)^.

Apesar dos benefícios trazidos com a inserção da amamentação como medida não farmacológica para redução da dor durante a vacinação, como a redução do tempo de choro e da frequência cardíaca, além de promover a aproximação da mãe com o lactente^(10)^, apenas um pequeno número de profissionais implementa essa técnica na rotina dos serviços de saúde^(11)^. Muitos ainda têm receio de que a criança se engasgue, broncoaspire ou vomite; porém, não há evidências na literatura até o momento que identifiquem esse fenômeno^(12)^.

Em virtude do contexto apresentado, o Ministério da Saúde (MS) do Brasil emitiu uma nota técnica em outubro de 2021 (Nota Técnica Nº 39/2021)^(13)^ que endossa a amamentação como uma medida não farmacológica para a redução de dores e desconfortos de crianças durante a aplicação de vacinas injetáveis. Essa técnica é balizada em estudos internacionais e nacionais que avaliaram a efetividade do processo de amamentação para redução da dor em lactentes^(3,14)^. No entanto, ainda há lacunas na literatura quanto ao momento de início da amamentação para favorecer a redução da dor frente à aplicação da vacina, com estudos avaliando a amamentação somente antes^(9,15)^ ou antes, durante e após a amamentação^(3,16)^, mas sem comparação em relação ao período mais indicado para a realização da amamentação.

Ao considerar que estudos randomizados e controlados são necessários para avaliar a eficácia e segurança da amamentação para procedimentos dolorosos^(10)^, em especial com abordagem do melhor momento para a intervenção^(9)^, em termos de minutos diferentes antes, durante e depois das vacinações^(14)^, os objetivos dessa pesquisa foram analisar o efeito da amamentação na redução da dor induzida pela vacina Pentavalente em lactentes e identificar o intervalo de tempo da amamentação necessário para sua ação antinocicepção.

## MÉTODO

### Tipo de Estudo

Ensaio clínico randomizado em *cluster*, realizado com três grupos paralelos, sem mascaramento. O estudo foi relatado com base no *Consolidated Standards of Reporting Trials* (CONSORT) *for Randomized Trials of Nonpharmacologic Treatments* e apresenta Registro Brasileiro de Ensaios Clínicos, com identificador primário: RBR-9vh37wr.

### Local

Os dados foram coletados em salas de vacinação, localizadas nos municípios de Floriano-PI e Barão de Grajaú-MA, Brasil. Optou-se por incluir apenas Unidades Básicas de Saúde (UBS) da zona urbana, uma vez que as salas de vacinação da zona rural nesses municípios não apresentam funcionamento em todos os dias úteis. As 16 UBS foram escolhidas de forma aleatória, por meio de randomização no site www.random.org.

### População e Critérios de Seleção

A população foi composta por 90 binômios (mãe-lactente) randomizados em 3 *clusters*: Grupo Intervenção 1 (GI1 - ­composto por binômios que realizaram o processo de amamentação cinco minutos antes da vacinação); Grupo Intervenção 2 (GI2 - composto por binômios que realizaram o processo de amamentação cinco minutos antes e durante a vacinação); e Grupo Controle (GC - composto por binômios que não realizaram o processo de amamentação).

Os critérios de inclusão para as mães foram: idade igual ou superior a 18 anos; que estavam realizando aleitamento materno (AM); com vestimenta que possibilitasse a amamentação. Para os lactentes, determinou-se: idade gestacional de 37–42 semanas; sem malformações congênitas visíveis e/ou referidas pela mãe; com necessidade de vacinação Pentavalente; e com faixa etária entre dois meses e dois meses e 29 dias.

Os critérios de exclusão foram: lactentes que não recebiam o AM direto da mama; que fizeram uso de analgésicos nas últimas 48 horas antes da vacinação; que estavam agitados antes da vacinação; que apresentaram histórico de hipersensibilidade a qualquer componente do imunobiológico e/ou outras contraindicações estabelecidas pelo MS^(17)^. Ainda, para os lactentes dos grupos intervenção, estabeleceu-se como critério a recusa ou dificuldade no processo de amamentação. Enfatiza-se que, dentre os grupos, os lactentes não precisaram ser do mesmo sexo, raça ou peso.

### Definição da Amostra

O cálculo amostral ocorreu a partir da fórmula para estudos com comparação entre grupos, considerando os seguintes parâmetros: nível de significância ou erro tipo I de α = 0,05, com 1 – α / 2 = 1,96, erro tipo II de b = 0,1, 1 – b = 0,90, tamanho do efeito ou d (µ1 - µ2) = 2,3 e desvio padrão (S1 = 0,4, S2 = 1,6) com base em estudo anterior^(15)^. A partir desses valores, obteve-se tamanho amostral de 9 indivíduos para cada grupo (controle e intervenção), o que totalizou o mínimo de 27 participantes para integrarem a amostra do estudo. Com vistas a ampliar a capacidade analítica, optou-se por continuar a coleta de dados, o que determinou uma amostra de 90 participantes: 30 no GI1; 30 no GI2; e 30 no GC.

### Coleta de Dados

A coleta de dados foi realizada apenas pela pesquisadora assistente, no período de agosto de 2022 a dezembro de 2023, de forma concomitante em todas as UBS alocadas. Foi utilizada randomização por *cluster*, na qual cada conglomerado tratou de um dos 16 subgrupos de participantes, com a inclusão de 6 no GI1, 5 no GI2 e 5 no GC. Para tal, foi utilizada lista dos 16 subgrupos, ordenados em sequência de indicação fornecida pelas Secretarias Municipais de Saúde, e a sequência numérica obtida no site determinou randomicamente quais foram alocados no GC e nos GI. Os participantes foram incluídos em cada grupo à medida que eram recebidos para realização da vacinação nas UBS, distribuídas em cada grupo.

No desenvolvimento da etapa de campo, primeiramente em uma sala privativa, forneceram-se orientações às mães participantes acerca dos objetivos, procedimentos, riscos e benefícios da pesquisa. Em seguida, aplicou-se um instrumento de caracterização socioeconômica, obstétrica e dos aspectos relativos à amamentação, utilizado em estudos anteriores^(18,19)^. Por fim, procedeu-se à execução da intervenção específica para cada grupo da amostra. Cabe mencionar que não foi possível relacionar apenas um profissional para realizar a administração das vacinas. Com isso, os profissionais das 16 UBS participantes do estudo receberam treinamento prévio, ministrado pela equipe de pesquisadoras, para padronização das técnicas e procedimentos relacionados ao processo de vacinação.

A vacina escolhida para o estudo foi a Pentavalente, vacina adsorvida difteria, tétano, *pertussis*, hepatite B (recombinante) e Haemophilus influenzae B (conjugada) apresentada sob a forma líquida em frascos multidose. Foi administrada a dose 1 (um) das 3 (três) doses estabelecidas pelo Programa Nacional de Imunizações do Brasil. Esse imunobiológico é considerado um dos que mais ocasionam dor, sendo o componente pertussis/coqueluche o principal responsável por ações reatogênicas, como vermelhidão, inchaço e dor no local da injeção^(20)^.

A técnica de administração da vacina Pentavalente foi unificada para os três grupos, conforme orientações do MS presentes no Manual de Normas e Procedimentos para Vacinação – MNPV^(17)^, com destaque para os seguintes aspectos: a vacina foi conservada entre +2 ºC e +8 ºC (sendo ideal +5 ºC), pois o congelamento provoca formação de agregados e aumenta o risco de reações; o volume da dose foi de 0,5 ml, administrado por via intramuscular profunda, no músculo vasto lateral da coxa esquerda; a agulha foi adequada ao ângulo de administração conforme a massa muscular do lactente a ser vacinado; foi utilizada seringa de 1 ml e agulha de tamanho 20 mm de comprimento e 5,5 dec/mm de calibre.

Tanto as mães dos GI como do GC seguraram a criança no colo durante a vacinação, realizada por profissional de enfermagem treinado, posicionado em frente à mãe. A pesquisadora assistente foi posicionada lateralmente ao binômio durante o procedimento, conforme estudo anterior semelhante^(3)^.

Durante o procedimento de vacinação da Pentavalente foi aplicada a Escala comportamental validada FLACC, desenvolvida para avaliação da dor em crianças entre dois meses e sete anos^(21)^. A escala apresenta cinco categorias de avaliação de acordo com o significado das iniciais da escala: Face (*face*), Pernas (*legs*), Atividade (*activity*), Choro (*cry*) e Consolabilidade (*consolability*). Cada categoria pode ser pontuada em uma escala de zero a dois e ter um resultado variando de 0–10, em que zero é considerado relaxado ou confortável, 1–3 desconforto pequeno, 4–6 dor moderada e 7–10 desconforto severo. Em 2008, Silva e Thuler^(22)^ realizaram a tradução para a língua portuguesa do Brasil e adaptação cultural da escala, obtendo resultados satisfatórios.

### Intervenção

A principal variável de desfecho foi a redução da dor nos lactentes vacinados. Como variável de desfecho secundário, foi determinado o melhor intervalo de tempo necessário da amamentação (somente antes ou antes e durante o processo de vacinação) para ação antinocicepção. A intervenção foi realizada tanto no GI1 como no GI2. Para os binômios alocados no GI1, foi solicitado que as mães, ainda na sala privativa e em uma cadeira confortável, amamentassem o lactente por 5 minutos antes da vacinação, tempo que foi monitorado com uso de cronômetro. Enfatizou-se que o monitoramento seria iniciado a partir do momento em que houvesse a comprovação de que o lactente estava sugando efetivamente, avaliado a partir dos pontos-chave estabelecidos pelo MS para determinação da pega adequada: 1. Mais aréola visível acima da boca do bebê; 2. Boca bem aberta; 3. Lábio inferior virado para fora; 4. Queixo tocando a mama. Também foi identificada a deglutição (visível e/ou audível)^(23)^, o que identificava a sucção nutritiva, ou seja, que o lactente estava deglutindo leite materno. Em seguida, as mães foram orientadas a suspender a amamentação durante a vacinação.

Para os binômios alocados no GI2, solicitou-se que as mães, ainda na sala privativa e em uma cadeira confortável, amamentassem o lactente por 5 minutos antes da vacinação, conforme os critérios estabelecidos para o GI1. Posteriormente, as mães foram orientadas a suspender a amamentação somente enquanto era administrada a Vacina Rotavírus Humana, seguindo as orientações do MS do Brasil, que determina que vacinas orais devem ser administradas antes de vacinas injetáveis^(13)^. Imediatamente, foram orientadas a reiniciar o processo de amamentação e mantê-lo durante todo o procedimento da vacinação da Pentavalente, que foi encerrado com uma suave compressão no local da administração da vacina com algodão seco. Os grupos intervenção (GI1 e GI2) tiveram os dados coletados em aproximadamente 30 minutos.

Os participantes alocados no GC foram aqueles binômios que receberam o cuidado habitual do serviço de saúde e, portanto, sem realização do processo de amamentação antes ou antes e durante o procedimento de vacinação. A duração da coleta de dados do GC foi de aproximadamente 15 minutos. Ao final, todas as participantes receberam um impresso com a Nota Técnica N°39/2021 do MS.

### Análise e Tratamento dos Dados

Os dados foram digitados em dupla entrada e armazenados em planilhas no Microsoft Excel® versão 2011. Em seguida, foram processados e analisados no programa do pacote estatístico *Statistical Package for Social Sciences for Windows* (SPSS) (2009) versão 20.0. Foi realizada a análise descritiva dos dados por meio das frequências absoluta e relativa, assim como da medida de tendência central, média, mediana e do desvio padrão. Para verificar a homogeneidade dos dados nos GI e GC, foi utilizado o teste Exato de Fisher para variáveis qualitativas. Em seguida, a distribuição das variáveis de desfecho foi avaliada por meio do teste de Kolmogorov-Smirnov. Como sua distribuição não era normal, os resultados de testes não paramétricos, como o teste de Kruskal-Wallis e o teste de comparações múltiplas de Dunn, foram relatados para comparações entre grupos e pares, respectivamente. O nível de significância de 5% e o intervalo de confiança de 95% foram adotados para todos os testes.

### Aspectos Éticos

O estudo atendeu às Resoluções nº 466/12 e nº 580/2018 do Conselho Nacional de Saúde. Foi aprovado pelo Comitê de Ética em Pesquisa do *Campus* Amílcar Ferreira Sobral da Universidade Federal do Piauí no ano de 2023, sob Parecer 6.083.435. Ressalta-se que a participação foi condicionada à assinatura do Termo de Consentimento Livre Esclarecido pela participante.

## RESULTADOS

Foram elegíveis para avaliação 95 binômios. Destes, 05 não atenderam aos critérios de inclusão, sendo 90 aleatorizados em três grupos: amamentação antes da vacinação (30 participantes), amamentação antes e durante a vacinação (30 participantes) e controle (30 participantes). O fluxograma de rastreamento dos participantes inclusos no estudo é apresentado na Figura 1.

**Figura 1:** Diagrama representativo do fluxo de participantes em cada fase do estudo, adaptado do *Consolidated Standards of Reporting Trials* (CONSORT). Floriano-PI/Barão de Grajaú-MA, Brasil, 2022-2023 (n = 90).

Não houve diferença estatisticamente significativa entre os grupos de intervenção e controle quanto às características socioeconômicas em termos de faixa etária, etnia, escolaridade, estado civil, renda familiar, e realização de atividade remunerada (p > 0,05); de modo semelhante, o perfil obstétrico não diferiu significativamente entre os grupos no que concerne à idade gestacional, realização de consulta pré-natal, orientações recebidas sobre amamentação, complicações na gravidez e tipo de parto (p > 0,05). Os grupos também apresentaram homogeneidade quanto aos aspectos relativos ao processo de amamentação: se o lactente foi amamentado imediatamente após o parto e se houve contato pele a pele ao nascer (p > 0,05) (Tabela 1).

**Tabela 1:** Caracterização das mães quanto aos dados socioeconômicos, obstétricos e relativos à amamentação nos grupos controle e intervenção. Floriano, PI/Barão de Grajaú, MA, Brasil 2022/2023 (n = 90).

Variáveis	Grupo Intervenção 1 n* (%)	Grupo Intervenção 2 n* (%)	Grupo Controle n* (%)	Média ± Dp^†^	p-Valor^‡^
	Caracterização socioeconômica				
**Faixa Etária**				28,48 ± 6,63	0,273^§^
18 a 28 anos	18 (60,0%)	17 (56,7%)	12 (40,0%)		
29 a 43 anos	12 (40,0%)	13 (43,3%)	18 (60,0%)		
**Etnia**					0,206^§^
Branca	5 (16,7%)	1 (3,3%)	1 (3,3%)		
Parda /Negra	25 (83,3%)	29 (96,7%)	29 (96,7%)		
**Escolaridade**					0,954^§^
Ensino Fundamental Incompleto a Ensino Médio Incompleto	7 (23,3%)	8 (26,7%)	9 (30,0%)		
Ensino Médio Completo a Ensino Superior Completo	23 (76,7%)	22 (73,3%)	21 (70,0%)		
**Estado Civil**					0,070^§^
Solteira	8 (26,7%)	14 (46,7%)	6 (20,0%)		
Casada	22 (73,3%)	16 (53,3%)	24 (80,0%)		
**Renda familiar** ^¶^					0,285^§^
Até 2 salários-mínimos	27 (90,0%)	22 (73,3%)	26 (86,7%)		
>2 salários-mínimos	3 (10,0%)	8 (26,7%)	4 (13,3%)		
**Realização de atividade remunerada**					0,421^§^
Não	20 (66,7%)	18 (60,0%)	23 (76,7%)		
Sim	10 (33,3%)	12 (40,0%)	7 (23,3%)		
**Caracterização obstétrica**
**Idade gestacional**				39,03 ± 1,35	0,184^§^
37**–**39	20 (66,7%)	16 (53,3%)	23 (76,7%)		
40**–**42	10 (33,3%)	14 (46,7%)	7 (23,3%)		
**Realização de consulta pré-natal**	**–**
Não	**–**	**–**	**–**		
Sim	30 (100%)	30 (100%)	30 (100%)		
**Recebeu orientações sobre a amamentação**					0,554^§^
Não	8 (26,7%)	6 (20,0%)	10 (33,3%)		
Sim	22 (73,3%)	24 (80,0%)	20 (66,7%)		
**Apresentou complicações na gravidez**					0,114^§^
Não	28 (93,3%)	22 (73,3%)	22 (76,7%)		
Sim	2 (6,7%)	8 (26,7%)	7 (23,3%)		
**Tipo de parto**					0,876^§^
Cesáreo	22 (73,3%)	22 (73,3%)	20 (66,7%)		
Normal	8 (26,7%)	8 (26,7%)	10 (33,3%)		
**Lactente foi amamentado imediatamente após parto**					0,451^§^
Não	14 (46,7%)	12 (40,0%)	9 (30,0%)		
Sim	16 (53,3%)	18 (60,0%)	21 (70,0%)		
**Houve contato pele a pele ao nascer**					0,774^§^
Não	16 (53,3%)	13 (43,3%)	13 (43,3%)		
Sim	14 (46,7%)	17 (56,7%)	17 (56,7%)		

* = amostra;

^†^Dp = Desvio padrão;

^‡^ *p-*Valor = nível de significância;

^§^Teste exato de Fisher;

^¶^Salário-mínimo vigente = R$ 1.212, Brasil, 2022.

Quanto à média de dor dos lactentes nos respectivos grupos, em GI1 (amamentados apenas antes da vacinação) foi de 6,06 ± 1,25 (Mediana = 6); em GI2 (amamentados antes e durante a vacinação) a média de dor foi de 3,83 ± 1,23 (Mediana = 4); e no grupo controle, a média de dor dos lactentes foi de 7,43 ± 1,30 (Mediana = 7,5). As comparações pareadas mostraram que há efeito da amamentação na redução da dor [X^2^(2) = 52,238; p < 0,05) entre os grupos (Tabela 2).

**Tabela 2:** Análise de comparação entre os grupos utilizando a escala de FLACC. Floriano, PI/Barão de Grajaú, MA, Brasil, 2022/2023 (n = 90).

Escore de dor – Escala FLACC									p-Valor^1^
Grupo intervenção I (n = 30)			Grupo intervenção II (n = 30)			Grupo controle (n = 30)			0,000		
Min	Máx	Mediana (IQR) / Média ± DP	Min	Máx	Mediana (IQR) / Média ± DP	Min	Máx	Mediana (IQR) / Média ± DP	
4	9	6 (2) / 6,06±1,25	2	7	4 (1) / 3,83 ± 1,23	5	10	7,5 (2,25) / 7,43 ± 1,30	

^1^Kruskal-Wallis.

Conforme mencionado anteriormente na subseção de análise dos dados, realizamos post hoc utilizando o teste de comparações múltiplas de Dunn considerando a Correção de Bonferroni igual a 0,0167. Primeiro, nos dois testes, os GI1 e GI2 foram comparados com o grupo controle nas respostas comportamentais de dor durante a vacinação Pentavalente. Os resultados mostraram que apenas o GI2 apresentou pontuação significativamente (p < 0,05) menor nas respostas comportamentais de dor em comparação com o grupo controle. Em outras palavras, amamentação 5 minutos antes e durante o processo de vacinação foi mais eficaz que a amamentação apenas antes para diminuir as respostas comportamentais de dor dos lactentes durante a vacinação pentavalente (Tabela 3).

**Tabela 3:** Análise de comparação entre pares de grupos utilizando a escala FLACC. Floriano, PI/Barão de Grajaú, MA, Brasil, 2022/2023 (n = 90).

Amostra 1 - Amostra 2	Teste estatístico	Desvio padrão	p-Valor^1^	p-Valor^1^ intergrupo
GI2 - GI1	29,567	6,67	0,000	0,000
GI2 - GC	47,783	6,67	0,000	0,000
GI1- GC	18,217	6,67	0,006	0,019

^1^Teste de comparações múltiplas de Dunn.

A classificação da dor também foi avaliada ­considerando-se os grupos investigados, conforme descrito na Tabela 4. Verifica-se que nenhum lactente do GI1 apresentou dor leve; 20 apresentaram dor moderada e 10, forte. No GI2, 14 lactentes apresentaram dor leve, 14 apresentaram dor moderada e apenas 2 mostraram dor forte. Por fim, no grupo controle, nenhum lactente apresentou dor leve, ao passo que 8 apresentaram dor moderada e 22, dor forte (p = 0,000).

**Tabela 4:** Análise de comparação da classificação de dor. Floriano, PI, Brasil, 2023.

Grupo intervenção I			Grupo intervenção II			Grupo controle			p-Valor^1^
Leve	Moderada	Forte	Leve	Moderada	Forte	Leve	Moderada	Forte	**0,000**
**–**	20	10	14	14	2	**–**	8	22	

^1^Teste exato de Fisher.

## DISCUSSÃO

Os resultados demonstraram que os lactentes pertencentes ao GI2 (amamentados com sucção nutritiva, ou seja, deglutindo leite materno, cinco minutos antes e durante a administração da vacina Pentavalente) obtiveram melhor resposta comportamental de redução da dor, observada por meio da escala FLACC, quando comparados aos demais grupos. Apesar de se observar redução da dor entre os lactentes do GI1 (amamentados apenas antes da vacinação), os níveis de desconforto ainda são elevados.

O escore médio de dor no GI2 foi de 3,83 (±1,23), enquanto no GI1 e GC foi de 6,06 (±1,25) e 7,43 (±1,30), respectivamente. A diferença foi estatisticamente significativa (p = 0,001) apenas no GI2, o que indica que, para redução significativa da dor dos lactentes durante a vacinação, a amamentação precisa ser realizada em momento oportuno.

A indicação da ação antinocicepção da amamentação no presente estudo soma-se ao conjunto da literatura que apoia essa prática durante procedimentos de rotina, como vacinação, para reduzir a dor entre lactentes. Uma revisão de escopo com objetivo de examinar como foram conduzidas pesquisas sobre o manejo não farmacológico em crianças com dor relacionada à vacinação no ambiente de saúde recomendou, como primeira alternativa, a amamentação, depois soluções adocicadas e, por fim, a sucção não nutritiva para reduzir a dor relacionada ao procedimento em recém-nascidos e lactentes^(7)^.

A partir das evidências apresentadas na Nota Técnica nº 39/2021^(13)^ acerca das intervenções não farmacológicas para redução da dor durante o procedimento da vacinação em criança que esteja em aleitamento materno, recomenda-se que os serviços de saúde favoreçam e apoiem a presença dos pais ou responsáveis durante e após o procedimento de vacinação e incentivem a lactante em amamentar a criança imediatamente antes e durante a administração de vacinas injetáveis.

Quanto ao momento oportuno de início e duração da amamentação, os resultados encontrados apresentam semelhança com estudo realizado com o objetivo de determinar a eficácia da amamentação para o alívio da dor durante a vacinação dos bebês, que foram amamentados dois minutos antes e durante o procedimento; tal estudo demostrou que a amamentação reduziu significativamente o nível de dor^(24)^. Pesquisa desenvolvida com o objetivo de identificar o efeito da amamentação no nível de intensidade da dor da imunização em lactentes amamentados antes, durante e após a vacinação também concluiu que a amamentação tem efeito positivo altamente expressivo, estatisticamente significativo, como método não farmacológico na redução do nível de intensidade da dor entre lactentes^(16)^.

Diferentemente, em estudo com objetivo de investigar a eficácia da amamentação na redução da dor em recém-nascidos submetidos ao teste do pezinho, os pesquisadores argumentaram que não houve diferença significativa nos escores médios de dor durante a coleta de sangue do calcanhar de recém-nascidos durante a amamentação nos grupos de estudo e controle e reconheceram a possibilidade de que o intervalo de tempo que escolheram (dois minutos antes, com a interrupção da amamentação anterior a realização do procedimento doloroso) não tenha sido longo o suficiente para obtenção do efeito antinociceptivo do leite materno^(25)^.

Nesse sentido, evidencia-se que amamentar os lactentes cinco minutos antes e durante o procedimento de vacinação é suficiente para redução da dor, intervalo de tempo que também foi estabelecido em estudos prévios envolvendo administração das vacinas Hepatite B^(12)^ e Pneumocócica Conjugada^(14)^, nos quais houve redução da dor, o que mostra convergência com o resultado desta pesquisa.

A amamentação proporciona melhores respostas comportamentais à dor (reduz tempo de choro e pontuações de dor) durante a vacinação em comparação com ausência de manejo, água oral e outras intervenções, como carinho, glicose oral, anestésico tópico, massagem e sprays refrigerantes^(10)^. É possível afirmar que na amamentação uma rede complexa de componentes multifatoriais se integra, permitindo o alcance máximo da capacidade analgésica dessa prática. Infere-se que, desde o momento do preparo da mãe, quando esta coloca o lactente no colo para permitir o início da sucção não nutritiva, que é responsável por desencadear o reflexo de ejeção do leite, até o alcance da sucção nutritiva, fenômenos químicos e comportamentais convergem para gerar o relaxamento e alívio da dor do lactente.

Os mecanismos subjacentes ao efeito benéfico da amamentação contra a dor ocasionada pela vacinação ainda são indefinidos^(3)^. No entanto, foi constatado em estudo anterior que devido ao sabor doce da sacarose presente no leite humano e à estimulação oral-tátil da sucção não nutritiva, a serotonina e a endorfina são liberadas, produzindo um efeito analgésico que dura entre cinco e 10 minutos^(26)^. Esse fato associa-se à estimulação dos sentidos do lactente, através do cheiro do aroma materno^(27)^, escuta dos batimentos cardíacos^(28)^ e sensação tátil de contenção e proteção promovida pelo colo materno^(29)^.

Uma revisão do banco de dados Cochrane, que objetivou avaliar a eficácia da amamentação ou da suplementação do leite materno na redução da dor em neonatos, indicou que os componentes da amamentação que podem ser analgésicos incluem a presença de uma pessoa reconfortante (mãe), sensação física (contato pele a pele com a pessoa reconfortante), desvio de atenção/distração e doçura do leite humano (presença de lactose ou outros componentes)^(10)^.

Essa conjunção de mecanismos, que sugerem potencializar o efeito da amamentação na redução da dor gerada pela vacinação, explica os resultados do presente estudo que indicam a amamentação somente antes da vacinação como insuficiente agente antinociceptivo. Dessa forma, considera-se que diferentes intervenções não farmacológicas podem ter efeitos analgésicos coordenados e, portanto, recomenda-se essa combinação proporcionada pela amamentação para maximizar a analgesia frente à vacinação^(7)^.

Outros métodos não farmacológicos podem ser empregados para o manejo da dor em lactentes não amamentados submetidos a procedimentos dolorosos, os quais podem ser aplicados isoladamente ou associados, a saber: administração oral de soluções adocicadas, como sacarose, glicose e dextrose, em diferentes concentrações; sucção não nutritiva; Método Mãe Canguru e contato pele a pele; enfaixamento (*swaddling*); aplicação de vibração mecânica; massagem; contenção; posição de aconchego; entre outros.

Ressalta-se que, dentre os lactentes amamentados durante o procedimento de vacinação, nenhum apresentou complicações, como engasgo, tosse, aspiração ou cianose. Apenas uma criança do GI1 regurgitou após a administração da Vacina Rotavírus Humana, condição que pode estar associada à própria vacina^(17)^. A crença na possibilidade dessas intercorrências, muitas vezes verbalizadas pelos profissionais de enfermagem, são limitantes e podem impactar negativamente ao desencorajar a amamentação durante a vacinação para o alívio da dor do recém-nascido e do lactente^(30)^. Em uma revisão sistemática do banco de dados Cochrane, nenhum dos estudos incluídos relatou intercorrências relacionadas à amamentação durante procedimentos invasivos, sugerindo, então, que não há risco de efeitos adversos como os mencionados^(31)^.

O presente estudo possui resultados relevantes para os profissionais de saúde ao considerar que a amamentação constitui intervenção natural e desprovida de necessidade de instalação especial ou de investimentos financeiros. Portanto, esse método não farmacológico deve ser definitivamente implementado nas salas de vacinação para o manejo e controle da dor em lactentes. Cabe aos profissionais de saúde, com destaque para a equipe de enfermagem inserida nos serviços de atenção primária à saúde, em especial salas de vacinação, incentivarem a prática da amamentação durante procedimentos dolorosos, como a administração de vacinas injetáveis. Por fim, indica-se que a equipe de enfermagem empregue escalas de avaliação da dor na rotina de salas de vacina como instrumentos que podem avaliar a qualidade das experiências e a eficiência das suas abordagens.

Este é um estudo de centro único, cuja validade externa se restringe a uma região do país. Posteriormente, é necessário um estudo multicêntrico com amostra maior para confirmar os resultados da pesquisa. Outra limitação é o fato de que, devido à natureza das intervenções, não foi possível cegar os membros da equipe que as conduziram e os participantes. Além disso, a aplicação da escala FLACC, que avalia respostas comportamentais, embora validada, constitui outra limitação. Nesse sentido, sugere-se que em estudos futuros parâmetros objetivos sejam avaliados, como medidas fisiológicas. Apesar dessas limitações, a intervenção mostrou-se eficaz na análise.

## CONCLUSÃO

A amamentação realizada cinco minutos antes e durante a administração da vacina Pentavalente reduziu significativamente o escore de dor quando comparada à amamentação apenas antes do procedimento. Não foram identificados riscos capazes de superar os benefícios da amamentação durante sua realização; portanto, tal prática deve ser implementada na rotina das salas de vacinação, por se tratar de um método natural, acessível e exequível. Assim, endossa-se a Nota Técnica nº 39/2021 do Ministério da Saúde, ao passo que se sugere que o termo “imediatamente”, presente no documento, deve corresponder a cinco minutos antes da vacinação.
